# Lifestyle considerations in multiple myeloma

**DOI:** 10.1038/s41408-021-00560-x

**Published:** 2021-10-26

**Authors:** Yael N. Shapiro, Jeffrey M. Peppercorn, Andrew J. Yee, Andrew R. Branagan, Noopur S. Raje, Elizabeth K. O’ Donnell

**Affiliations:** 1 Massachusetts General Hospital, Boston, MA USA; 2 Harvard Medical School, Boston, MA USA

**Keywords:** Quality of life, Myeloma

## Abstract

As the prognosis for multiple myeloma (MM) has significantly improved and patients remain on therapy longer, there is a need for supportive care interventions to optimize patient quality of life (QOL) and functional status over the course of cancer treatment. MM is characterized by a significant symptom burden and a relatively lower QOL compared to other cancers. This review evaluates the role of healthy lifestyle behaviors in improving both the physical functioning and psychological well-being of the MM population. We (1) describe the current literature on physical activity, weight management, diet, sleep, and substance use in the context of MM, (2) present important considerations for incorporating lifestyle factors into clinical practice, and (3) identify directions for future research. Developing MM-specific guidelines for modifiable lifestyle changes that take into account both the length of treatment and the unique disease features (i.e. osteolytic lesions and anemia) may provide a promising path for improved patient QOL and functioning.

## Introduction

Multiple myeloma (MM) is the cause of 1% of cancer deaths globally and is the second most common hematological malignancy in the United States [1]. Over the past fifteen years, the availability of new effective drugs with acceptable short-term toxicities has modified the traditional treatment paradigm for MM such that patients are now treated continuously for their disease. These treatments have also increased the five-year relative survival to 55.6% [2]. The combination of prolonged survival and the continuous administration of maintenance medication and the resulting impact on quality of life (QOL) has highlighted the need to focus research efforts on addressing the long-term side effects, both physical and emotional, of MM therapy. It is essential that we develop supportive care interventions to ensure that patients’ QOL and functional status is maximized as they live longer with the chronic burdens of their cancer therapies.

Studies have demonstrated high symptom burden and low reported QOL among MM patients [3]. MM patients may suffer from more symptoms in comparison to other hematological cancers [3–5]. In a recent study [6] of 16 095 cancer survivors, MM patients scored the lowest for both health-related QOL and physical functioning. As tremendous efforts are being placed on advancing MM pharmacological therapies and improving overall survival, there is an increasing need to examine ways in which lifestyle interventions can also be incorporated into care. It is critical to evaluate the role of healthy behaviors such as physical activity, sleep, weight management, nutrition, and reduction of substance use in enhancing QOL, mitigating disease- and treatment-related side effects, and improving long-term outcomes for MM patients.

This review investigates the role of physical activity, weight management, nutrition, sleep, and substance use in MM. We will review the literature on the relationship between these domains and MM and explore future directions for research.

## Physical activity

There is a growing field of research examining the potential positive impacts of exercise among adults with cancer. Evidence strongly suggests that physical activity is safe and effective as an adjuvant therapy both during and after cancer treatment. In 2010, the American College of Sports Medicine developed physical activity guidelines for cancer patients and survivors [7, 8], both increasing awareness about exercise as a form of therapy and highlighting its significance in health outcomes. While they urged cancer survivors to avoid inactivity and to stay as active as possible, they also emphasized the importance of modifying exercise based on disease- and treatment-specific complexities and limitations. They recommended engaging in aerobic activity of moderate to vigorous intensity for at least 150 min per week, resistance training for two or more days per week, and stretching exercises daily. These guidelines have served as a benchmark for exercise trials since. As evidence about physical activity and cancer continues to expand, with the number of randomized controlled exercise trials spiking to over 2,500 publications, the society convened in 2019 to revise the exercise guidelines [8]. The updated guidelines recommend moderate-intensity aerobic activity for at least 30 min, at least three times per week, and resistance training at least two times per week [8].

In a recent meta-analysis, exercise was shown to improve fatigue, muscle strength, aerobic fitness, QOL, anxiety, and self-esteem, particularly among patients with breast, lung, colon, and prostate cancers [9–16]. Another systematic literature review evaluated 10 studies of exercise interventions aimed at improving physical functioning and psychological well-being, specifically among patients with hematological malignancies. While none of the studies were found to be of high methodological quality, making firm conclusions difficult, these studies demonstrate the feasibility of conducting exercise interventions for hematological cancers [17]. Few studies, however, have specifically looked at exercise for MM patients [18].

MM may be especially debilitating compared to other forms of cancer, explaining low rates of physical activity and further complicating the prospect of incorporating exercise for this population. A pilot study [19] utilized cardiopulmonary exercise testing to evaluate the effect of autologous stem cell transplantation on MM patients’ cardiopulmonary fitness up to 17 months post-transplant. Exercise capacity, measured by peak oxygen consumption, was 38 ± 18% less than age- and sex- normative values and 6 min walk test 25 ± 13% less than predicted values. These results suggest that MM patients experience significant decreases in physical functioning extending long beyond initial treatment.

Furthermore, a low percentage of MM patients are physically active—it was found that only 6.8% and 20.4% (*p* < 0.001) of MM patients met the American College of Sports Medicine 2010 physical activity guidelines during active and off-treatment periods, respectively [20]. A cross-sectional study (*N* = 229) [21] was conducted with MM patients to compare levels of pre-diagnosis and post-diagnosis physical activity levels and to examine barriers and motivators for attending an exercise program. It was found that significantly more patients met the American College of Sports Medicine recommended guidelines of 150 min of moderate-intensity exercise pre-diagnosis (38.9%) versus post-diagnosis (20.1%) (*p* < 0.001) [7, 21]. Those who were physically active prior to diagnosis were found to be five times more likely to continue to meet the physical activity guidelines after diagnosis. The most commonly reported barriers to exercise were fatigue (37.8%), injuries (34.2%), pain (28.1%), coexisting health conditions (21.1%), and declines in physical functioning due to age (18.5%). Importantly, readily modifiable barriers including lack of knowledge about how to exercise safely (19.7%), low self-confidence in physical ability (17.1%), and fear of injury (16.2%), were also reported. Interestingly, interpersonal factors such as not having someone to work out with (10.1%), lack of time (10.1%), the high cost of exercise (9.2%), and nausea (7.9%), were less likely to be reported as barriers [21].

Craike et al. [21] also evaluated the likelihood of MM patients to participate in physical activity and found that 41.1% of MM patients would participate in an exercise program specifically designed for their disease; this significantly lags behind survivors of other cancers, such as bladder cancer (81.1%) [22], lymphoma (81%) [23], and kidney cancer (70%) [24]. It is important to acknowledge that MM is more commonly diagnosed in an older age population, with the median age of diagnosis at roughly 70 years [25]. The predominate older age incidence presents a unique challenge for the adoption of exercise as a component of MM therapy.

Although most of the research is centered on risk and prevention of MM, there are few interventional studies that directly look at the incorporation and impact of physical activity during the active MM disease course.

### Physiological effects

Several studies have focused on the physiological benefits of exercise, such as change in body composition, reduced fatigue, and improved blood cell counts, among individuals living with and undergoing treatment for MM (Table 1a). Overall, the available data demonstrates that exercise is feasible, safe, and may improve physical function, fatigue, mood, and sleep [26–28]. Further, it was found that exercise has profound physiologic benefits for patients such as reducing the discomfort and time burden of treatments such as transfusions [29].

**Table 1:** a Physiological effects of exercise on MM patients. b Psychological effects of exercise on MM patients.

Author, year	Study design, sample	Study purpose	Main findings
(a)			
Coleman et al., 2003 [26]	Pilot/feasibility RCT, *N* = 24	To evaluate the feasibility of a home-based exercise program for MM patients receiving high-dose chemotherapy and ASCT • Program consisted of stretching, strength, resistance, and aerobic exercises • Length of program and session frequency not reported	• Individuals assigned to the intervention group gained an average of 0.40 kg in lean body weight per month while those assigned to the usual care group experienced an average decline of −0.44 kg in lean body weight per month, a statistically significant difference of 0.84 kg per month (*p* < 0.01) • A tailored exercise program for MM patients undergoing treatment is feasible and may reduce fatigue and mood disturbances while improving sleep
Coleman et al., 2008 [29]	RCT, *N* = 135	To examine the effects of aerobic and strength resistance training combined with EPO therapy on transfusions, stem cell collections, transplantation recovery, and MM treatment response among MM patients receiving high-dose chemotherapy and ASCT • Home-based individualized exercise program, incorporating stretching, aerobic, and strength resistance exercises 15-week program (session frequency not reported)	• The exercise group had significantly fewer red blood cell transfusions and fewer attempts at stem cell collection (*p* < 0.025) • Recovery time and treatment response were not significantly different after transplantation between the exercise and usual care groups • Exercise has profound physiologic benefits for patients, reducing the discomfort and time burden of treatments such as transfusions
Groeneveldt et al., 2013 [27]	Single-arm pilot study, *N* = 37	To assess the feasibility of a 6-month home-based exercise program on MM patients and its effects on QOL and physiological outcomes • Program consisted of stretching, aerobic, and resistance exercises • Six-month program (3 times/week) • Three months were supervised and three months were at home	• High attendance in supervised classes (87%) and high adherence rates in home-based classes (73%) throughout the study duration • There was an increase in upper and lower limb muscle strength (*p* < 0.001) at the completion of the program • Fatigue improved from baseline to 6 months (*p* < 0.01 for both, one-way repeated measures ANOVA) • An exercise program is feasible and safe for MM patients
Servadio et al. 2020 [28]	Observational, *N* = 175	To assess whether exercise is associated with health-related quality of life (HRQOL) in MM patients	• Physically active patients reported fewer treatment-related side effects (*p* = 0.001)

(b)			
Courneya et al., 2000 [30]	Prospective, *N* = 25	To examine the correlation between exercise and QOL among cancer patients who had received high-dose chemotherapy and autologous bone marrow transplantation	• Exercise during hospitalization was significantly correlated with QOL, across multiple dimensions (*p* < 0.001) such as physical well-being, psychological well-being, depression, and anxiety
Jones et al., 2004 [20]	Retrospective/ observational, *N* = 88	To investigate the association between exercise and QOL in MM patients (at three time periods: prediagnosis, active treatment, and off-treatment)	• Only 6.8 and 20.4% of the MM survivors met national exercise guidelines during active treatment and off-treatment periods, respectively • Exercise, particularly moderate intensity, both during active and off treatment was found to be positively associated with QOL and its subdomains (social well-being, emotional well-being, functional well-being) (all *P* < 0.05), except for physical well-being
Groeneveldt et al., 2013 [27]	Single-arm pilot study, *N* = 37	To assess the feasibility of a 6-month home-based exercise program on MM patients and its effects on QOL and physiological outcomes • Program consisted of stretching, aerobic, and resistance exercises • Six-month program (3 times/week)	• Improvements in QOL from baseline to 6 months (*p* < 0.01)
Servadio et al., 2020 [28]	Observational, *N* = 175	To assess whether exercise is associated with health-related quality of life (HRQOL) in MM patients	• Physically active patients reported a higher HRQOL (*p* = 0.001) • Exercise was not associated with psychological outcomes (ie anxiety and depressive symptoms) (anxiety: *p* = 0.139; depressive symptoms: *p* = 0.073)

### Psychological effects

Exercise has also been found to play a significant role in improving mood and QOL in MM patients. The available data (Table 1b) suggest that exercise is positively associated with QOL and its subdomains (social well-being, emotional well-being, functional well-being) and psychological distress [20, 27, 28, 30].

While physiological and psychological parameters are distinct, it is important to note that they work in tandem and studies often show an improvement in both with the incorporation of exercise into the treatment regimen. Psychological factors such as the psychosocial domains of QOL may even serve as independent predictors of overall survival among MM patients [31]. Though limited, this research points to the instrumental role that both mind and body can potentially play in the MM disease course. Further research of the physiological and psychological benefits of exercise for MM patients is needed.

### Treatment considerations

Lytic lesions develop in approximately 80% of MM patients [32], raising concerns for clinicians and patients regarding incorporating and encouraging exercise as a critical therapy during a MM diagnosis. Skeletal injuries, the ongoing risk of pathological fractures, chronic bone pain, and sarcopenia make this patient population particularly challenging candidates for exercise interventions [27, 32]. Fear of fractures is a widespread concern and causes many MM patients to give up sports and other leisure activities [33]. But, research, although limited for MM, points to the many potential physiological and psychological benefits of safely incorporating exercise with cancer care, such as improved QOL, blood cell counts, body composition, strength, mood, and fatigue.

Educating and training clinicians to engage in conversations about exercise will encourage MM patients to feel more comfortable and confident participating in physical activity. Jones et al. [34] (*N* = 311) found that most cancer patients prefer that their oncologist initiate discussions about exercise. Despite this, only 28.4% of the study participants reported that their oncologist initiated a conversation about exercise during a treatment consultation, and almost 60% reported that exercise was not discussed at all. Oncologist-initiated conversations not only resulted in a stronger belief in exercise (*p* < 0.001) among the cancer patients but also in longer (*p* < 0.07) and more frequent (*p* < 0.001) physical activity participation [34]. Oncologists may also need guidance in terms of appropriate exercises. Fear of further bone damage in the setting of the lytic disease may inhibit providers from making recommendations about physical activity. A multidisciplinary approach that includes physical therapy and physical medicine and rehabilitation may assist oncologists in providing exercise recommendations. In addition, bone-directed therapy with either zoledronic acid or denosumab, is recommended for all MM patients to reduce the risk of skeletal-related events.

There is also a need for randomized controlled trials in the MM population so that we can best guide patients about how to incorporate a safe and effective exercise routine into their treatment regimen. With further research, we will learn more about the effects of exercise on the various parameters of MM and be able to curate individualized exercise routines, tailored to each patient’s unique preferences, treatment history, and complications.

### Body composition and diet

In 2014, an estimated 640 million adults were suffering from obesity globally, which is six times the number in 1975 [35]. Obesity is the second leading cause of cancer and excess body fat has been linked to 13 types of cancer, including MM [35–38]. In 2003, a groundbreaking prospective study of 900,053 adults found that men and women who were very obese (body mass index (BMI) of at least 40 kg/m^2^) had a 52 and 62% higher rate of death from all cancers, respectively, than men and women of normal weight [39].

Not only can obesity lead to the development of cancer, provocative evidence suggests that obesity may impact survival once patients develop MM. Given a 5-year survival rate of only 54% [40], a potentially modifiable factor such as obesity must be explored. The aforementioned study found a significant positive linear trend in death rates with increasing BMI among MM patients (*p* = 0.002) [39]. Another meta-analysis of ten prospective studies found that with each increase in BMI of 5 kg/m^2^ (over 25 kg/m^2^), there is an increased incidence of MM (relative risk = 1.1, *p* < 0.0001) for both men and women [41]. Evidence has even supported the role of excessive weight and obesity in the transformation of monoclonal gammopathy of undetermined significance (MGUS) to MM (*p* = .002) [42]. The mechanism of obesity’s impact on disease development and outcomes is unknown.

However, the association between BMI and MM incidence and outcomes suggests a need to consider the role of weight loss and nutrition in disease management. Few research studies have looked at physiological parameters such as body composition and weight specifically during the active disease course. We can, however, highlight research that evaluates the impact of MM and its treatments on body composition and the influence of body composition on MM progression and outcomes. Nutrition-related recommendations for MM patients will also be explored.

### MM effects on weight

MM is characterized by a wide array of disease- and treatment-specific complications that contribute to weight management challenges. Bone pain, the most common symptom seen in the MM population, prevents many patients from engaging in physical activity and thereby leads to muscle wasting, physical deconditioning [19], and often subsequent weight gain. Pervasive sleep disturbances can also contribute to weight gain. The MM treatment course is distinguished by continuous high-dose corticosteroid usage. This prolonged exposure makes MM patients particularly vulnerable to lean muscle loss, gain of visceral adipose tissue, increased appetite, weight gain, hyperglycemia, and hyperlipidemia; all of these issues are modifiable or potentially preventable side effects that can affect long-term survival and QOL. The combination of chronic corticosteroid use, advanced age, and physical deconditioning due to inactivity contributes to the widespread weight issues seen in MM patients.

Several studies have described these physical changes, providing us with a further lens into patients’ weight-related challenges during MM. Greenfield et al. [43] examined the long-term endocrine, metabolic, and nutritional status of 32 advanced, aggressively treated MM patients. Endocrine abnormalities were prevalent, consisting of hypothyroidism (9%), hypogonadism (65% of males), and high prolactin levels (19%), which are all linked to impaired body composition (e.g. fat gain, muscle loss).

The modality chosen for the assessment of body mass is an important consideration. In one study, total body dual-energy x-ray absorptiometry scans found sarcopenic-obesity in 65% of participants [43]. In contrast, when BMI was assessed for the same population, only 43% were classified as obese (BMI > 30 kg/m^2^). While BMI, which considers both weight and height to assess overall body fat, serves as a helpful base for comparison, it does not consider muscle or bone composition, both relevant factors for MM patients. To better capture and understand how weight contributes to both the incidence of MM and disease outcomes, there is a need to integrate valid and reliable standardized modes of body composition assessment into clinical care and research studies.

### Impact of weight on MM outcomes

Various studies have evaluated how weight modulates the active MM disease course. One study [44] focused on the correlation of body composition with disease activity, adverse events, and treatment response in 108 newly diagnosed MM patients. Patients received a whole-body low-dose CT before induction therapy to measure body fat composition such as visceral adipose tissue and subcutaneous adipose tissue. No correlation was found between body fat composition and disease activity parameters (e.g. M-protein, lactate dehydrogenase, bone marrow cells). A reciprocal correlation was found between adverse cytogenetics and visceral adipose tissue, respectively (gain 1q21: *p* = 0.009 and *p* = 0.021; *t*(4;14): *p* = 0.038 and *p* = 0.042). There was also a significant reciprocal correlation between treatment response and abdominal (*p* = 0.03) and pelvic (*p* = 0.035) visceral adipose tissue. Interestingly, BMI was not found to be significantly correlated with treatment response or cytogenetics, again stressing the value in evaluating body composition rather than BMI alone.

The impact of obesity on treatment, specifically high-dose chemotherapy and autologous stem cell transplantation, has also been studied. Vogl et al. [45] evaluated the outcomes of 1087 MM patients who had undergone autologous stem cell transplantation. They categorized patients into four weight classes, normal (18.5 ≤ BMI < 25, *n* = 292), overweight (25 ≤ BMI < 30, *n* = 472), obese (30 ≤ BMI < 35, *n* = 198), and severely obese (BMI ≥ 35, *n* = 125), and found no significant differences between obese and non-obese patients on outcomes such as progression-free survival, overall survival, or non-relapse mortality. Dosing strategies of melphalan varied though, emphasizing the need to develop standardized optimal dosing guidelines for overweight and obese MM patients to maximize efficacy while reducing toxicities.

To our knowledge, there are currently no interventional studies that evaluate the role of weight gain and loss on active MM. Most of the research is centered on the prevention of MM and examining excess weight and obesity as risk factors for disease.

### Research on nutrition in MM

With our increasing knowledge of the detrimental impacts of excess body fat on both the incidence and progression of cancers such as MM, nutrition must be examined. The American Cancer Society [46] recommends a healthy diet centered around plant-based foods, with at least 2−3 cups of vegetables and 1½ to 2 cups of fruits each day. Whole grains are preferred to refined grain products, and processed meat and red meat consumption should be limited. Controlling caloric intake and engaging in regular physical activity are also recommended for cancer patients and survivors to maintain a healthy weight [46]. Beyond these general cancer guidelines, there is limited data about specific diets for MM patients.

Although the research on nutrition during MM is limited, various studies have highlighted the nutritional deficiencies frequently seen in MM patients. Greenfield et al. [43] found that 37.5% of participants were vitamin D deficient (<30 nmol/L) while an additional 21.8% had inadequate levels (30–50 nmol/L). Twenty-five percent were also found to be folate deficient and 6% had reduced levels of vitamin B12. Another retrospective chart review study (*N* = 78) [47] found that 14.1% of MM patients had folate deficiency. These parameters are captured in routine MM screenings and represent common baseline deficiencies for this population. The specific nutritional profile of MM patients, though, has not been studied.

The scarcity of research centered around diet and MM outcomes does not accurately reflect the prevalence of patient concerns. MM patients commonly complain of weight gain and are interested in nutritional guidance to self-manage and improve their health. Cho et al. [1] examined the supportive care needs of 141 patients with MM and found that education on healthy foods was a priority for patients, following requests for information on future disease outcome and honest medical staff explanations. Patients with MM are interested in how their diets can be modified to improve their disease course and long-term health outcomes.

### Treatment considerations

Although the research is limited, obesity is a modifiable risk factor for MM and thus must be addressed. Both MM-specific weight and nutrition guidance should be provided as components of a treatment plan. The development of specific diets for MM patients can also hopefully improve outcomes such as minimizing disease- and treatment-related side effects. Patients may also experience changes in appetite, smell, and taste from MM and its treatments, which can lead to further weakness and fatigue in addition to baseline disease levels [48]. Thus, guidance about recommended foods and meal schedules may assist MM patients in managing a healthy diet. In addition, medications, such as dexamethasone, may contribute to weight gain and change in body composition such as loss of lean muscle mass and gain of visceral fat. Dose reductions and discontinuation of dexamethasone during maintenance are recommended when possible to mitigate these side effects. To develop tailored guidelines, future studies are needed to examine common metabolic and nutritional profiles of MM patients. Nutrition is modifiable and may have the potential to influence both short- and long-term outcomes.

### Sleep

Poor sleep quality is one of the most common complaints among cancer patients, including those with MM. Studies show that sleep disturbances are present in about 30−75% of the cancer population, disproportionately impacting cancer patients at twice the rate of the general population [49, 50].

Adequate sleep quality plays a central role in maintaining individuals’ physiological and mental health. Poor sleep quality and duration have been linked with a range of outcomes such as the increased risk of infection, cardiovascular disease, diabetes, cognitive impairment, metabolic dysfunction, and mood-related disorders such as depression and anxiety [51, 52]. The disturbed mood has been found to be associated with fatigue severity, which might be further exacerbated by the use of MM medications such as corticosteroids [53]. Sleep deprivation can also impact appetite regulation. Various studies have found that sleep deprivation is associated with a decrease in leptin levels and an increase in ghrelin levels [54–56]. As the national trend shows a decrease in sleep duration, there has been a simultaneous sharp increase in obesity, perhaps suggesting that the two are linked [57]. Increased daytime napping also often reduces physical activity and can lead to physical deconditioning and weight gain [53].

Strong evidence indicates that sleep is also closely tied to QOL. A longitudinal study (*N* = 397) [58] found that individuals who experienced frequent sleep disturbances scored significantly lower on all QOL subscales of the Medical Outcomes Survey Short Form (SF)−36 compared to those without sleep problems (OR: 1.71−18.32). Effects may be further pronounced when compounded with cancer and its many disease- and treatment-related symptoms.

### MM-specific risks for sleep disturbances

Sleep disturbances among MM patients are likely caused by a multitude of factors such as pain, medication, and affective disorders such as depression and anxiety. Bone pain is one of the defining symptoms of MM, with almost 80% of newly diagnosed patients reporting bony lesions [25]. Prolonged exposure to high-dose corticosteroids such as dexamethasone is a key component of all MM combination regiments and has been linked to insomnia, daytime sleepiness, and fatigue [59]. Peripheral neuropathy has also been associated with treatments such as thalidomide and bortezomib [60, 61]. The associated sensory symptoms such as feelings of numbness, tightness, burning sensation, and sharp pain are often worse at night, further contributing to sleep disturbances [62]. Iron-deficiency anemia, renal failure, and peripheral neuropathy are major risk factors for secondary sleep disorders such as restless legs syndrome and periodic limb movements [59]. The potent combination of side effects from the disease itself and its associated treatments contribute to the difficulties that MM patients experience in maintaining optimal sleep duration and quality. Identification of these barriers to sleep is paramount in devising strategies for reducing sleep disturbances.

The heavy psychological burden associated with the diagnosis of an incurable cancer such as MM may have a profound impact on patients’ sleep and overall QOL. Depression and anxiety are strongly correlated with sleep disturbances before, during, and after cancer treatment [53, 63, 64]. Molaissiotis et al. [65] examined the unmet supportive care needs and psychological well-being of 132 MM patients and 93 of their partners. Tiredness (40.7%), pain (35.9%), and insomnia (32.3%) were the most frequently reported symptoms. Twenty-seven percent of patients reported markers of anxiety and 25% of the patients reported markers of depression, highlighting the pervasive emotional burden of the disease. Further studies are needed to evaluate the correlations between psychological disorders and sleep quality, specifically among MM patients.

### Sleep in MM

Despite the extensive risks and prevalence of sleep disturbances, there is limited research exploring the sleep experiences of MM patients. Most of the relevant research has focused on ways in which sleep is affected during MM treatment, as opposed to its role in modulating the progression of active disease.

Various studies have examined the effects of the MM disease course on patient’s sleep quality and duration (Table 2). These studies provide insight into the sleep profile of MM patients and emphasize the need to address sleep disturbances in this population. Sleep improvement can help patients to maintain their daily routine, in addition to improving fatigue, mood, physical functioning, and QOL, in the midst of a lifelong disease. More research specifically focused on MM patients’ experiences will shed light on the prevalence of sleep-related problems and the need for sleep-promoting interventions.

**Table 2:** Sleep in MM patients.

Author, year	Study design, sample	Study purpose	Main findings
Parsons et al., 2019 [33]	Qualitative, *N* = 32	To examine the lived experiences and treatment priorities of patients with relapsed or refractory MM	• All participants indicated fatigue as a major concern during their disease course, reporting that extreme exhaustion interfered with many aspects of daily living such as concentrating on tasks and going to work
Coleman et al., 2011 [53]	Qualitative, *N* = 187	To describe fatigue, sleep, pain, mood, and performance status among patients with newly diagnosed MM	• Participants’ sleep was characterized by: • Increased daytime sleep duration (19% of total average sleep time) • Frequent awakenings (mean was 12 times, SD = 5.7) • Low sleep efficiency (time asleep while in bed at night, mean 80%, SD = 14.2)
Potter et al., 2016 [85]	Retrospective, *N* = 38	To evaluate the incidence of sleep-disordered breathing	• 68% of patients were taking narcotic pain medications and 30% were taking sleep medications • High levels of cardiac complications were also observed, such as valvular dysfunction (70%), diastolic dysfunction (41%), left ventricular hypertrophy (37%), and cardiomyopathy (7%). • 95% of patients had clinically significant sleep-disordered, with the majority (83%) being diagnosed with obstructive sleep apnea (OSA)

Sleep-disordered breathing has also been found to be associated with MM (Table 2). This limited research emphasizes the importance of monitoring sleep and addressing sleep problems throughout the course of cancer treatment for MM patients.

### Treatment considerations

Growing research has examined the importance of sleep on our physiological and mental health, potentially impacting our QOL, physical functioning, mood, fatigue, and long-term health outcomes. While numerous studies outline how sleep is affected during MM treatment, there is no research to our knowledge that evaluates the role of sleep nor the impact of improving sleep on the active MM disease course and its progression. Sleep measures, both objective (e.g. polysomnography, actigraphy, bispectral index) and subjective (e.g. Pittsburgh Sleep Quality Index, Insomnia Severity Index, Epworth Sleepiness Scale, Consensus Sleep Diary), need to be included in studies [66]. This will help us understand the importance of sleep, the impact of MM treatments on sleep, and the repercussions of sleep loss on patients’ daily functioning and QOL. Further research on sleep in MM will help clinicians to provide more effective interventions, tailored to their disease-specific medications and complications.

Clinicians should encourage good sleep hygiene practices such as limiting daytime naps, maintaining a consistent sleep schedule, avoiding caffeine, alcohol, or foods or drinks with a high sugar content at night, reducing noise and light in one’s bedroom, and engaging in relaxing activities before bedtime. Strategies to counterbalance sleep interfering side effects of corticosteroids, such as taking the dose in the morning, can also prove valuable [67]. There are various nonpharmacological interventions that show promising effects in reducing fatigue and alleviating sleep disturbances for cancer patients, such as exercise [8, 9, 62, 68], massage therapy [69, 70], cognitive behavioral therapy for insomnia (e.g. cognitive restructuring, stimulus control, sleep restriction, relaxation training, sleep education, and hygiene) [71–73], relaxation therapy, and meditation [49], but have not been well integrated into routine clinical practice [50]. Pharmacological interventions, such as dose-reductions of steroids, can be considered if sleep problems persist. In addition, several classes of sleep-inducing medication can be considered but are typically intended for short-term or periodic use, and can have adverse effects, including dependence [74]. Managing symptoms and identifying potential sleep disorders can help alleviate sleep disturbances and potentially improve mood, physical functioning, and QOL.

### Substance use

Substance use is another important lifestyle consideration in the context of MM. Tobacco smoking is a leading cause of preventable deaths, accountable for nearly one in five deaths in the US and six million deaths globally each year. Multiple studies over the past few decades have found no association between cigarette smoking and MM risk [75], but little is known about how the bone marrow microenvironment changes with tobacco metabolites.

Alcohol use has also been explored as a risk factor for MM. Alcohol is considered a carcinogen, accounting for about 6% of all cancers and 4% of all cancer deaths in the US [76, 77]. It has been associated with various cancers such as of the throat, liver, and breast. Interestingly, some studies suggest that alcohol consumption may have a protective effect against MM development [78, 79]. Most of these studies, though, had a small sample size and limited statistical power. A pooled analysis [80] of 1567 cases and 7296 controls found that ever drinking (current) was associated with a decreased risk of MM for both men (OR = 0.72, 95% CI 0.59–0.89) and women (OR = 0.81, 95% CI 0.68–0.95) compared to the never drinking group. This association was seen across different alcoholic beverages such as beer, wine, and liquor and did not differ when adjusted for smoking status, BMI, or education. Further studies are needed to elucidate the biological mechanisms underlying the relationship between alcohol and MM, as well as, to evaluate the dose-response associations with frequency and duration of consumption. Light intake of alcohol may improve immune response and the DNA repair system while heavy intake may impair the immune system and make the body more susceptible to infection. The American Cancer Society recommends abstinence, but if one chooses to drink alcohol, intake should be limited to one drink per day for women and two drinks per day for men [76].

Aside from the aforementioned research on risk, there are no studies, prospective or retrospective, to our knowledge that examine the relationship between smoking and alcohol use and active MM. MM patients struggling with this incurable, highly fatal disease are eager to treat their disease but also want to enjoy life beyond their diagnosis. Both smoking and alcohol may likely serve as outlets for patients to relieve stress and thus, their relationship to the MM disease course must be examined.

The United States Drug Enforcement Administration lists marijuana and its cannabinoids as Schedule I controlled substances, so they cannot legally be prescribed, possessed, or sold under federal law [81]. Many states, however, permit marijuana use for medical conditions such as cancer, and social acceptance is increasing, especially within the cancer community. A study [82] found that 18.3% of patients at a community oncology clinic reported using cannabis in 2017. MM patients may use marijuana to alleviate disease- and treatment-related symptoms such as nausea, loss of appetite, pain, neuropathy, and anxiety.

It should be noted, though, that there are possible harms to marijuana use. While providing a sense of euphoria, marijuana also lowers one’s control over movement, causes disorientation, and may trigger unpleasant thoughts or anxiety [81]. Similar to smoking tobacco, smoking marijuana is particularly harmful as it spreads chemicals to both the user and others. Additionally, the levels of active compounds can vary greatly between marijuana plants, and thus, it is challenging to predict an individual user’s experience [81]. Lastly, it is possible to develop a dependence on marijuana.

Despite the current restrictions on cannabinoid research, there is intriguing evidence on the therapeutic value of marijuana use for MM patients. Morelli et al. [83] found that the combination of cannabidiol and proteosome inhibitor bortezomib was more effective in inducing MM cell death than bortezomib alone. A recent study by Nabissi et al. [84] found a synergistic effect between Δ [9]-tetrahydrocannabinol, cannabidiol, and cytotoxic agent carfilzomib, resulting in strong anti-cancer activity; it reduced MM cell viability by inducing autophagic-cell death and inhibited MM cell migration by down-regulating expression of chemokine receptor CXCR4 and the CD147 plasma membrane glycoprotein.

The American Cancer Society [81] supports the need for further scientific research on cannabinoid use among cancer patients. Medical decisions regarding symptom and pain management should be made between the patient and physician, taking into account benefits and harms, patient preferences and values, and relevant laws and regulations [81]. Further research will help us to better understand the mechanisms of marijuana and its potential role in MM management.

## Discussion

Lifestyle interventions, including tailored physical activity and nutrition interventions, may serve as valuable routes for MM patients to manage their health and perhaps improve their physical functioning, fatigue, QOL, psychological distress, and long-term health outcomes. Guidelines have been established for the general cancer population (Table 3), but the associations between improved weight management, nutrition, sleep, substance use, and disease outcomes have not yet been adequately elucidated for MM patients. Although questions and concerns about weight and nutrition are among the most common for patients, limited research is available to guide the development of MM-specific guidelines. Our review provides a foundation for developing tailored guidelines that take into account the unique disease features of MM. Further studies of specific lifestyle interventions such as exercise and dietary regimens must be explored. Developing MM-specific guidelines for modifiable lifestyle changes that take into account both the length of treatment and the unique disease features (i.e. osteolytic lesions and anemia) may provide a promising path for improved patient QOL, function, and ultimately, MM outcomes.

**Table 3:** Lifestyle guidelines for adult cancer survivors*.

Physical activity [7, 8, 46, 86]	• Engage in regular physical activity • Avoid inactivity and return to exercise as soon as possible • Engage in at least 150–300 min per week of moderate-intensity exercise (ie pilates, gardening, brisk walking) or 75 min per week of vigorous-intensity aerobic exercise (ie aerobic dance, running, hiking uphill) [46] • Engage in moderate-intensity aerobic activity for at least 30 min, at least three times per week, for at least 8–12 weeks and resistance training, at least two times per week [8] • Incorporate strength training exercises at least 2 days per week
Body composition and diet [46, 48]	• Achieve and maintain a healthy weight (BMI between 18.5 and 25 kg/m^2^) • If overweight or obese, limit consumption of high-calorie foods and beverages and increase physical activity • Maintain a diet that is high in vegetables, fruits, and whole grains • Limit intake of processed foods and drinks • Limit intake of red meats
Sleep [87–90]	• Maintain a regular sleep schedule of 7 h or more per night • Components of good sleep hygiene: • Limit daytime naps, stay active during day, maintain consistent sleep schedule, avoid caffeine, alcohol, or foods or drinks with a high sugar content at night, reduce noise and light in one’s bedroom, and engage in relaxing activities before bedtime • Cognitive behavioral therapy for insomnia (CBT-I) should be considered [9] • Exercise, relaxation therapy, massage therapy, and meditation may also help alleviate sleep disturbances • Sleep inducing medication may be considered, but typically for short-term or period use
Substance use [76, 77, 81, 91]	• Avoid smoking tobacco • Avoid drinking alcohol, if possible • Limit alcohol intake to one drink per day for women and two drinks per day for men • More scientific research is needed on cannabis use • Medical decisions about pain management should be made between the patient and physician, taking into account patient preferences and local regulations • Smoking or vaping of marijuana should be avoided due to the carcinogens in smoke

*Cancer type, stage, associated treatments, and comorbid conditions should be considered when making recommendations.
